# Identification of CD8^+^ T-Cell–Immune Cell Communications in Ileal Crohn's Disease

**DOI:** 10.14309/ctg.0000000000000576

**Published:** 2023-03-01

**Authors:** Han G. Duong, Eunice J. Choi, Paul Hsu, Natalie R. Chiang, Shefali A. Patel, Jocelyn G. Olvera, Yi Chia Liu, Yun Hsuan Lin, Priscilla Yao, William H. Wong, Cynthia S. Indralingam, Matthew S. Tsai, Brigid S. Boland, Wei Wang, John T. Chang

**Affiliations:** 1 Department of Medicine, University of California San Diego, La Jolla, California, USA;; 2 Department of Chemistry and Biochemistry, University of California San Diego, La Jolla, California, USA;; 3 Department of Medicine, Jennifer Moreno Department of Veteran Affairs Medical Center, San Diego, California, USA;; 4 Department of Cellular and Molecular Medicine, University of California San Diego, La Jolla, California, USA.

**Keywords:** single-cell RNA-sequencing, cell-cell communications, CD8^+^ T cells, Crohn’s disease, inflammatory bowel disease

## Abstract

Crohn's disease (CD) is a major subtype of inflammatory bowel disease (IBD), a spectrum of chronic intestinal disorders caused by dysregulated immune responses to gut microbiota. Although transcriptional and functional changes in a number of immune cell types have been implicated in the pathogenesis of IBD, the cellular interactions and signals that drive these changes have been less well-studied.

**METHODS:**

We performed Cellular Indexing of Transcriptomes and Epitopes by sequencing on peripheral blood, colon, and ileal immune cells derived from healthy subjects and patients with CD. We applied a previously published computational approach, NicheNet, to predict immune cell types interacting with CD8^+^ T-cell subsets, revealing putative ligand-receptor pairs and key transcriptional changes downstream of these cell-cell communications.

**RESULTS:**

As a number of recent studies have revealed a potential role for CD8^+^ T-cell subsets in the pathogenesis of IBD, we focused our analyses on identifying the interactions of CD8^+^ T-cell subsets with other immune cells in the intestinal tissue microenvironment. We identified ligands and signaling pathways that have implicated in IBD, such as interleukin-1β, supporting the validity of the approach, along with unexpected ligands, such as granzyme B, which may play previously unappreciated roles in IBD.

**DISCUSSION:**

Overall, these findings suggest that future efforts focused on elucidating cell-cell communications among immune and nonimmune cell types may further our understanding of IBD pathogenesis.

## INTRODUCTION

Inflammatory bowel disease (IBD) is a chronic intestinal disorder typically categorized as Crohn's disease (CD) or ulcerative colitis (UC) based on clinical, endoscopic, and histopathologic criteria (1). UC is limited to the colon, whereas CD can affect any part of the digestive tract. IBD is generally considered to result from dysregulated innate and adaptive immune responses to gut microbiota in genetically susceptible individuals, although genetic changes are neither necessary nor sufficient for the development of IBD (2).

The cellular and molecular basis of the IBDs is not fully understood, but a number of diverse immune and nonimmune cells have been implicated in its pathogenesis, such as epithelial cells (3), stromal cells (4), macrophages (5), innate lymphoid cells (6), and subsets of CD4^+^ and CD8^+^ T lymphocytes (7–10). Using traditional methods, such as bulk RNA sequencing, histopathology, and flow cytometry, along with newer methods, such as single-cell RNA sequencing and mass cytometry, many studies have sought to characterize changes in specific cell types within the blood and intestinal tissues of individuals with IBD (3,4,7–13). However, less is known about the cellular interactions and signals that drive observed numerical, phenotypic, transcriptional, and functional alterations of specific cell types.

In this article, we performed Cellular Indexing of Transcriptomes and Epitopes by sequencing (CITE-seq) (14), which enables measurement of the transcriptome and selected proteins in the same single cells. We applied CITE-seq to peripheral blood, colon, and ileal immune cells derived from healthy subjects and individuals with CD affecting the ileum. We annotated the cells using a published RNA-seq reference data set (15) and applied NicheNet, a computational approach that infers ligand-receptor relationships (16), to elucidate putative cell-cell communications (CCC) and downstream transcriptional changes. As a number of recent studies have revealed a potential role for CD8^+^ T-cell subsets in the pathogenesis of the IBDs (7–10), we focused our analyses on identifying the interactions of CD8^+^ T-cell subsets with other immune cells in the intestinal tissue microenvironment. We identified ligands and signaling pathways that have implicated in IBD, such as interleukin-1β (IL-1β), supporting the validity of the approach, along with unexpected ligands, such as granzyme B, which may play previously unappreciated roles in IBD. Overall, the study serves as an integrated transcriptomic/protein resource data set for future research by other investigators and indicates that efforts focusing on elucidating CCC may increase our mechanistic understanding of IBD.

## METHODS

### Human subjects

The Human Research Protection Programs approved the study. Intestinal biopsies and peripheral blood were obtained from patients undergoing colonoscopy after obtaining informed consent. Healthy individuals were undergoing colonoscopy as part of routine clinical care for colorectal cancer screening/surveillance or noninflammatory gastrointestinal symptoms that included constipation or rectal bleeding. Inclusion criteria included age over 18 years and absence of significant comorbidities or colorectal cancer. CD patients with active endoscopic ileal disease on minimal to no medical therapy were selected. Details of the study subjects are provided in Supplementary Digital Content (see Supplementary Table 1, http://links.lww.com/CTG/A920).

### Human peripheral blood mononuclear cell isolation

Blood was collected in BD Vacutainer CPT mononuclear cell preparation tubes (BD Biosciences) and centrifuged at 400*g* for 25 minutes. The buffy coat layer was removed, washed, and counted. Cells were resuspended in freezing buffer {10% (v/v) dimethylsulfoxide (Sigma-Aldrich), 40% (v/v) complete Roswell Park Memorial Institute (RPMI) 1640 medium (RPMI [Corning] +10% [v/v] fetal bovine serum [FBS, Life Technologies] +100-U/mL penicillin/100-μg/mL streptomycin [Life Technologies]), 50% (v/v) FBS}, placed into a freezing container (Mr. Frosty), and stored at −80°C. Cells were recovered, washed, filtered, and labeled with anti-human CD45 (2D1) (BioLegend) for sorting. CD45^+^ immune cells were sorted on a FACSAria2 (BD Biosciences).

### Human intestinal cell isolation

Four intestinal biopsies were obtained with endoscopic biopsy forceps from the ileum and rectum and collected in separate conical tubes with Hank's buffered saline solution (HBSS, Corning). Intestinal biopsies were transferred into freezing buffer (10% [v/v] dimethylsulfoxide, 40% [v/v] complete RPMI, and 50% [v/v] FBS) and stored at −80°C. Biopsies were recovered, incubated in HBSS on a shaker, then incubated twice in HBSS + 5-mM dithiothreitol (Thermo Fisher Scientific) for 10 minutes on a rocker at 37°C, followed by a final HBSS wash. Intestinal biopsies were mechanically dissociated, then placed into a conical tube containing 10 mL of digestion mixture (complete RPMI + 1.5-mg/mL collagenase type VIII [Sigma-Aldrich] + 50-μg/mL DNase I [Roche]). The biopsies were digested in a shaking incubator (225 rpm) for 20 minutes at 37°C. The reaction was stopped with phosphate-buffered saline precooled to 4°C, filtered, and resuspended in fresh complete RPMI. Samples were incubated with BD Pharmingen Human BD Fc Block for 10 minutes to block nonspecific binding of the Fc receptor as described in the manufacturer's protocol. The 55-plex BD AbSeq antibody cocktail was generated using 2.6 μL of each antibody and brought to volume with BD buffer. Concurrently, samples were stained with anti-human CD45 antibody and a live/dead stain and incubated for 60 minutes on ice. CD45^+^ immune cells were sorted on a FACSAria2 and collected in complete RPMI media with 10% FBS.

### *In vitro* CD8 T-cell experiments

C57BL/6J mice were purchased from the Jackson Laboratory. Small intestine lymphocytes were harvested from uninfected C57BL/6J mice and subsequently plated and stimulated for measurement of cytokine production. Mice were housed under specific pathogen-free conditions in an American Association of Laboratory Animal Care–approved facility at UCSD, and all procedures were approved by the UCSD Institutional Animal Care and Use Committee. After euthanasia, small intestines were dissected, transected along the longitudinal axis, rinsed in calcium-free magnesium-free buffer (HBSS [Corning] + 10-mM N-2-hydroxyethylpiperazine-N'-2-ethanesulfonic acid [Fisher] + 2% [v/v] FBS [GenClone]), and shaken at 280 rpm with dithiothreitol (HBSS [Corning] + 10-mM HEPES [Fisher] + 10% (v/v) FBS [GenClone] + 1-mM dithiothreitol [Thermo Fisher Scientific]) at 37°C for 30 minutes. The lymphocytes were pelleted from supernatant and then underwent Percoll (Sigma) density gradient centrifugation to produce a lymphocyte-enriched layer. Cells were then resuspended in T-cell media (Iscove's Dulbecco's Modified Eagle Medium [Gibco] + 10% [v/v] FBS [Gibco] + 2-mM L-glutamine [Gibco], 100-units/mL penicillin, 100-μg/mL streptomycin [Gibco] + 50-μM β-mercaptoethanol [Gibco]) for stimulation. Lymphocytes from individual mice were aliquoted into a 96-well plate and then incubated for 4 hours at 37°C with 5% CO_2_ in the presence of Protein Transport Inhibitor Cocktail at 1x dilution (brefeldin A 10.6 μM, monensin 2 μM, eBioscience), phorbol myristate acetate 10 ng/mL (Sigma-Aldrich), and ionomycin 500 ng/mL (Sigma-Aldrich). Approximately 1 ng/mL of carrier-free IL-1β, granzyme B (GZMB), or IL-18 (BioLegend) were added to corresponding wells in triplicate. After stimulation, cells were stained with Fixable Viability Dye eFluor 780 (eBioscience) and fluorescently conjugated antibodies anti-mouse CD3, anti-mouse CD8α, anti-mouse CD8 β, and anti-mouse CD121a antibodies (Biolegend), followed by fixation with Foxp3/Transcription Factor Staining Buffer set (eBioscience), and subsequent staining with anti-mouse interferon γ (IFNγ), anti-mouse IL-2, and anti-mouse tumor necrosis factor (TNF) antibodies (Biolegend). Flow cytometry was performed with a NovoCyte Flow Cytometer (Agilent), and downstream analysis was performed using FlowJo v10.8.2 (FlowJo, LLC) and Prism v9.4.1 (GraphPad).

### 10× Genomics library preparation and sequencing

Cells were washed and resuspended in phosphate-buffered saline + 0.04% (w/v) bovine serum albumin (UltraPure BSA; Thermo Fisher Scientific) per the manufacturer's guidelines. Single-cell libraries were prepared according to the protocol for 10× Genomics for Single-Cell 3' Gene Expression. Approximately 20,000 sorted CD45^+^ cells resuspended at a final concentration of 4,000 cells/μL were loaded and partitioned into Gel Bead-in-EMulsions. The 10× Genomics cDNA generation protocol was modified to include the BD AbSeq PCR1 primer. After polymerase chain reaction amplification, the fractions containing the AbSeq oligos were processed through a separate library construction workflow. This AbSeq protocol generates libraries directly after cDNA amplification, eliminating intermediate steps as described in the 10x Genomics protocol. The quality of the sample fragments was determined by the High-Sensitivity D5000 and D1000 ScreenTape assay (Agilent Technologies). The scRNA and protein libraries were pooled at equimolar amounts for a final 10-nM concentration and sequenced on a NovaSeq 6000 S4 flow cell (Illumina).

### Computational analyses

Raw sequencing data were processed using the CellRanger pipeline (10x Genomics). Samples were further processed using Seurat v4.1.1 (17). Low-quality (e.g., dead) cells and potential doublets were filtered with the following parameters: <200 features, >10% mitochondrial features, and >2,500 features. antibody-derived tag and RNA assays were normalized using the standard Seurat workflow of “NormalizeData,” “FindVariableFeatures,” “ScaleData,” and “RunPCA.” Cell types were assigned using SingleR v.1.4.1 (18) and verified with canonical protein markers. Briefly, the log-normalized expression matrices were used as the inputs and Monaco labels served as the reference for label transfers. Label transfers were made using default singleR parameters. Revised “fine” labels were binned according to Supplementary Digital Content (see Supplementary Table 3, http://links.lww.com/CTG/A922).

Many algorithms are available to infer CCC using single-cell gene expression data (19–21). NicheNet is a CCC workflow that uses downstream differentially expressed genes (DEGs) in receiver cells to infer key ligand-receptor pairs from senders and receivers, respectively. A benefit of NicheNet is that the algorithm explains DEGs in receiver cells to predict key ligand-receptor pairs rather than only differentially expressed (DE) ligands and receptors. We opted to use the most updated version of NicheNet, Differential NicheNet, to prioritize both upstream ligands and receptors.

Briefly, the objective of NicheNet is to explain downstream DEGs in receiver cells by ranking upstream ligands and receptors in senders and receivers. All senders and receivers required > 25 identified cells within their respective tissue (e.g., Ileum_condition1_cell1>25 and Ileum_condition2_cell2>25). First, DE between the 2 cohorts' senders and receivers were used to define DE of ligand:receptor pairs using the Wilcoxon test. Niches were split by cell type, so large-cell populations would not dominate condition-specific DE analyses. The ligand and receptor must also be expressed by >10% of the cell population to qualify as a pairwise ligand-receptor to explain downstream DEGs. Then, ligand activities were calculated, and active-ligand targets were inferred. Ligand activities represent how well the ligand predicts observed changes in gene expression in receiver cells. Finally, ligand-receptor and ligand-target links were prioritized and the most important pairs per niche were visualized. For each receiver population of each condition, the top 15 prioritized ligand-receptor pairs were visualized as Circos plots. For ligand-target visualization, targets were required to share the same highly ranked ligand-receptors. To maximize the variety of sender-ligand pairs and to ensure the most confident ligand targets were visualized, the top 2 ligand-sender cell pairs with highest ligand target weights were visualized, where a higher ligand-target weight indicates higher confidence in that pair.

### Pathway enrichment analyses

The top 50 prioritized ligand-receptor pairs were inputs for ligand-receptor pathway analysis. For ligand-target pathway analysis, the ligand-targets were required to share the same highly ranked ligand-receptors upstream of targets. The top 2 ligand-sender pairs with the highest ligand-target weights served as pathway analysis inputs.

## RESULTS

**Figure 1.**
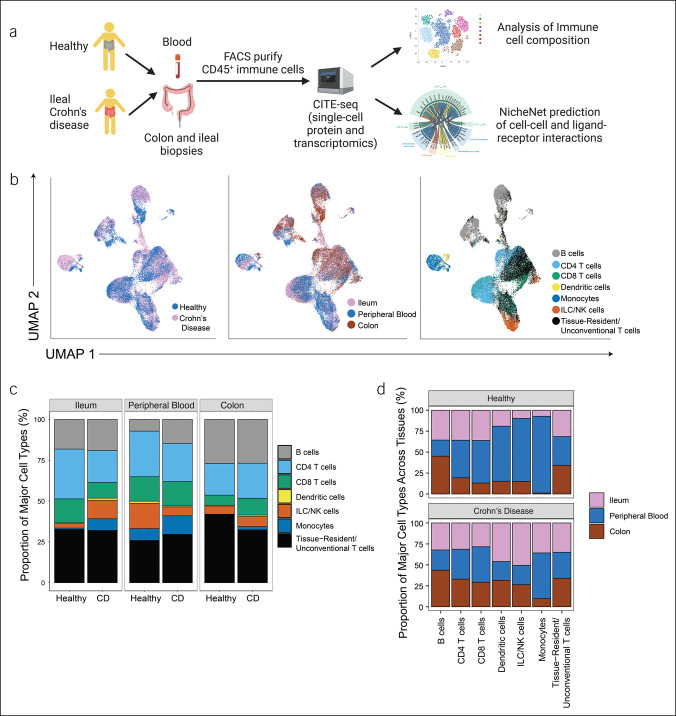
CITE-seq analyses of immune cells during health and Crohn's disease (CD). (**a**) Experimental design. Immune cells from blood, colon, and ileum derived from healthy subjects and individuals with CD were stained with the BD AbSeq antibody cocktail and fluorescently labeled antibodies, isolated by FACS, and prepared for CITE-seq using the 10× Genomics Chromium platform. Created with BioRender.com. (**b**) UMAP plots colored by health vs CD (left), tissue compartment identity (middle), or major immune cell type (right). (**c**, **d**). Proportions of each major immune cell type in each anatomic compartment from healthy subjects vs patients with CD. CITE-seq, Cellular Indexing of Transcriptomes and Epitopes by sequencing; UMAP, Uniform Manifold Approximation and Projection.

We obtained ileal and rectal mucosal biopsies along with peripheral blood from 7 healthy subjects and 9 patients with active ileal or ileocolonic CD (see Supplementary Table 1, http://links.lww.com/CTG/A920). Cells from mucosal biopsies and peripheral blood were processed into single-cell suspensions, fluorescence-activated cell sorting (FACS)-purified on the basis of CD45, a pan-immune cell marker, and processed for CITE-seq using the 10x Genomics Chromium platform (Figure 1a). Antibodies targeting 55 proteins were selected for inclusion in the BD AbSeq antibody panel (Table S2, http://links.lww.com/CTG/A921).

Uniform Manifold Approximation and Projection analyses revealed that immune cells from healthy subjects clustered distinctly from those derived from patients with CD (Figure 1b, left panel). Furthermore, immune cells from the peripheral blood generally clustered distinctly from those in the 2 intestinal tissue compartments (Figure 1b, middle panel), as has been previously observed (7,22). We first broadly annotated the immune cells into 7 major immune types (Figure 1b, right panel): B cells, CD4^+^ T cells, CD8^+^ T cells, dendritic cells, monocytes, innate lymphoid cells/natural killer cells (ILC/NK cells), and tissue-resident/unconventional T cells. Overall, B and T cells (including CD4^+^, CD8^+^, and tissue-resident/unconventional lymphocytes) were the most abundant immune cell types annotated in the data set (Figure 1c), and all immune cell types were identified in varying proportions across the intestinal tissues and peripheral blood (Figure 1d).

To annotate immune cells in greater detail, we used a published bulk RNA-seq reference data set in which the authors FACS-purified 29 immune cell subsets from the peripheral blood to define transcriptional signatures (15). Because these immune cell subsets were derived only from the peripheral blood, we revised the annotations of these subsets to account for cells in the tissues that exhibit transcriptional signatures similar to those of cells circulating in the peripheral blood (see Supplementary Table 3, http://links.lww.com/CTG/A922). For example, the original annotation “NK cells” was revised to “ILC/NK cells” because NK cells are considered a subset of the innate lymphoid cell (ILC) family and exhibit transcriptional similarities (23).

**Figure 2.**
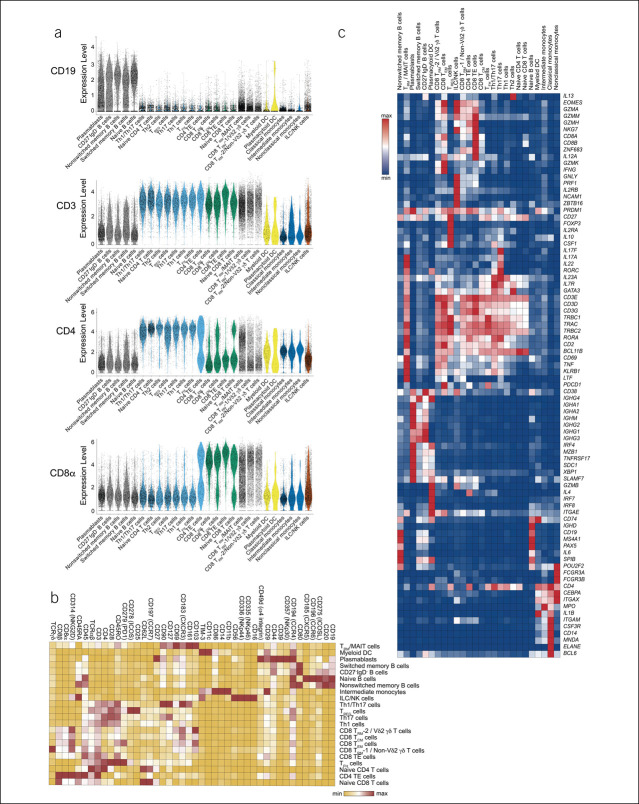
Expression of canonical genes and proteins in annotated immune cell subsets. (**a**) Expression of selected proteins in each of the 22 annotated immune cell subsets, represented as violin plots, ordered, and colored by major immune cell type (gray, B cells; light blue, CD4 T cells; green; CD8 T cells; black outline, tissue-resident/unconventional T cells; yellow, myeloid cells; blue, monocytes; brown, ILC/NK cells). (**b**) Relative expression of selected proteins included in the CITE-seq antibody panel, represented as a hierarchically clustered summary heatmap; columns represent individual proteins and rows represent each of the 22 annotated immune cell subsets. (**c**) Relative expression of selected canonical genes, represented as hierarchically clustered summary heatmaps; rows represent selected genes and columns represent each of the 22 annotated immune cell subsets. CITE-seq, Cellular Indexing of Transcriptomes and Epitopes by sequencing.

We confirmed the accuracy of the revised annotations using the protein and single-cell RNA-sequencing data. For example, protein expression of CD19, a canonical B-cell marker, was highest in the 5 B-cell subsets compared with other immune cell subsets (Figure 2a). Conversely, protein expression of CD3, a canonical T-cell marker, was highest in CD4^+^, CD8^+^, and tissue-resident/unconventional T-cell subsets. Moreover, cells annotated as CD4^+^ T-cell subsets generally expressed higher levels of CD4 protein, whereas cells annotated as CD8^+^ T-cell subsets generally expressed higher levels of CD8α protein. We observed that CD8^+^ and CD4^+^ tissue-resident memory (T_RM_) T-cell subsets were grouped with mucosal-associated invariant T (MAIT) cells, whereas subsets of CD8^+^ T_RM_ cells were annotated with Vδ2 γδ T cells and non-Vδ2 γδ T cells (Figure 2b,c, see Supplementary Table 3, http://links.lww.com/CTG/A922). Thus, application of previously published reference data sets may provide a useful framework for broadly categorizing immune cell subsets.

Previous studies have reported changes in the absolute numbers and frequencies of CD8^+^ T-cell subsets in the context of UC and CD (7–10). To identify CCC that might regulate quantitative and qualitative changes in CD8^+^ T-cell subsets in health vs CD, we applied NicheNet, a previously published computational approach (16). NicheNet uses previous knowledge of ligand-receptor interactions and gene expression patterns to infer ligand-target-gene relationships (16). Overall, the approach involves the following steps: (i) Defining the “sender” cell types, “receiver” cell types, and gene set that is to be used to guide the selection of prioritized ligands; (ii) defining a set of potential ligands expressed by the sender cell types that are known to bind putative receptors expressed by receiver cell types; (iii) ranking ligands based on presence of transcriptional signatures downstream of the potential ligand, and (iv) inferring target genes in receiver cells based on specific ligand-receptor pairs. In this way, putative ligand-receptor pairs can be identified between sender-receiver cell types, and gene expression changes downstream of engagement of ligand-receptor interactions can be assessed.

To illustrate the potential utility of applying NicheNet to identify interactions of CD8^+^ T-cell subsets with other immune cells, we first designated 4 ileal T-cell subsets as receivers: CD8^+^ effector memory (T_EM_), T_RM_/MAIT, CD8^+^ T_RM_-1/Vδ2 γδ T cells, and CD8^+^ T_RM_-2/non-Vδ2 γδ T cells. Examination of the expression of genes encoding for cytokines, cytokine receptors, and cytolytic granules suggested that T-cell subsets derived from patients with CD exhibit more inflammatory features than the same subsets derived from healthy subjects. For example, T-cell subsets derived from patients with CD tended to express higher levels of genes encoding cytolytic granules, such as *PRF1*, *GZMA*, and *GZMB*, along with genes encoding inflammatory cytokines, such as *IFNG* and *IL26* (see Supplementary Figure 1, http://links.lww.com/CTG/A923). Conversely, genes encoding receptors for cytokines that regulate homeostasis of naive and memory T cells (24), such as *IL7R*, were downregulated in T-cell subsets derived from patients with CD (see Supplementary Figure 1, http://links.lww.com/CTG/A923).

**Figure 3.**
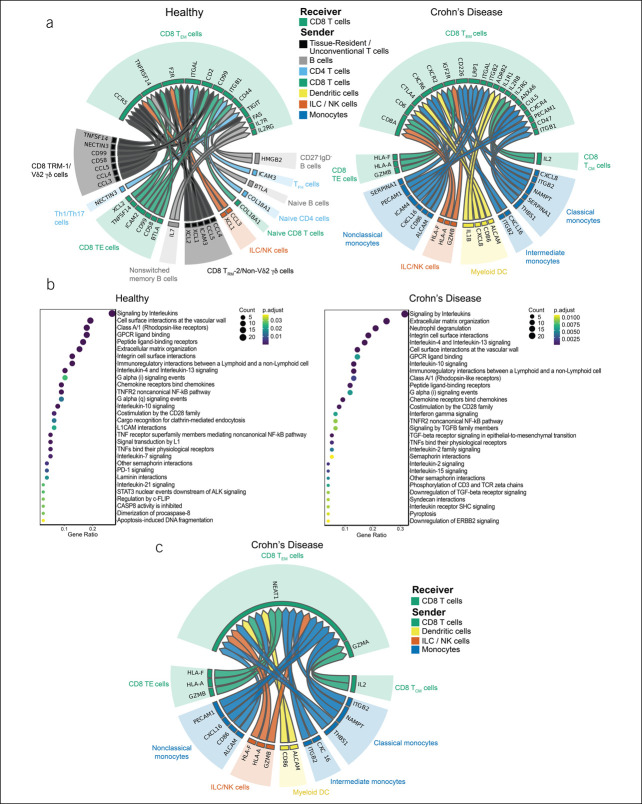
NicheNet analyses of ileal CD8 T_EM_-cell interactions with other immune cells in health vs Crohn's disease (CD). (**a**) Putative ligand:receptor pairs on “sender” immune cells and receiver cell (CD8^+^ T_EM_), represented as Circos plots, in healthy subjects (left) or patients with CD (right). Colors indicate major immune cell type; each major cell type is also indicated in the caption between the 2 Circos plots. Specific immune cell subsets are labeled around the Circos plot. (**b**) Pathway analyses of enriched genes downstream of predicted ligand:receptor pairs, represented as reactome plots, in healthy subjects (left) or patients with CD (right). (**c**) Genes predicted to be induced in CD8^+^ T_EM_ cells by predicted ligand:receptor interactions, represented as Circos plots, in patients with CD.

Next, we applied NicheNet to ileal CD8^+^ T_EM_ cells. Overall, we observed that ileal CD8^+^ T_EM_ cells from healthy subjects interacted with a broad array of immune cells, including CD8^+^ T cells, CD4^+^ T cells, γδ T cells, B cells, and ILC/NK cells (Figure 3a, left). By contrast, in the context of CD, ileal CD8^+^ T_EM_ cells interacted to a greater extent with monocyte subsets and myeloid dendritic cells (Figure 3a, right). Focusing on specific ligand-receptor interactions in the healthy state, we observed increased *IL7*-*IL7R* interactions. Moreover, *TNFSF14* (LIGHT/HVEML/CD258) on unconventional T cells and *BTLA* on B cells were predicted to bind to *TNFRSF14* (HVEM/CD270) on ileal T_EM_ cells (Figure 3a, left). Multiple cellular ligands have been discovered for herpesvirus entry mediator (HVEM), including LIGHT, B- and T-lymphocyte attenuator (BTLA), CD160, and lymphotoxin, resulting in different consequences depending on the cellular and environmental context (25). For example, HVEM-BTLA interactions typically result in an inhibitory immune response (26), whereas the interaction between HVEM and CD160 can inhibit activation of CD4^+^ T-cell subsets (27) or promote costimulatory effects, leading to cytokine production by splenic CD8^+^ T cells (28).

We observed a different set of predicted chemokine-chemokine receptor interactions in health vs CD, with *CCL3*, *CCL4*, and *CCL5* being more prominent in health and *CXCL8* and *CXCL16* being more prominent in patients with CD (Figure 3a). Moreover, in patients with CD, *IL1B* produced by myeloid dendritic cells was predicted to bind to its receptor *IL1R1* on CD8^+^ T_EM_ cells (Figure 3a, right); IL-1β–mediated signaling in CD8^+^ T cells has been reported to increase effector functions such as production of inflammatory cytokines and granzyme B (29). Examination of previously published gene expression data from patients with CD (12) confirmed that certain T-cell subsets expressed detectable levels of *IL1R1* (see Supplementary Figure 2A, http://links.lww.com/CTG/A923). Furthermore, *in vitro* experiments demonstrated that small intestine CD8^+^ T cells expressed IL-1R at the protein level and were capable of responding to exogenous addition of IL-1β by increasing production of IFN-γ (see Supplementary Figure 2B–F, http://links.lww.com/CTG/A923). *ALCAM*, which encodes for the activated leukocyte cell-adhesion molecule expressed on nonclassical monocytes and myeloid dendritic cells, was predicted to bind to *CD6*, a costimulatory molecule expressed on T cells, which has been shown to play an integral role in modulating T-cell activation, proliferation, and trafficking (30,31) (Figure 3a, right). IL-2, a cytokine known to promote T-cell proliferation, was predicted to be produced by CD8^+^ central memory (T_CM_) cells and bind to *ILRB* and *ILRG*, which encode components of the IL-2 receptor, in CD8^+^ T_EM_ cells derived from patients with CD. Finally, *GZMB* produced by other CD8 T cells and ILC/NK cells was predicted to bind to *IGF2R* on CD8^+^ T_EM_ cells (Figure 3a, right), which raised the intriguing possibility of CD8^+^ T-cell– and NK-cell-mediated killing of CD8^+^ T_EM_ cells in the context of CD, perhaps as a compensatory mechanism to attenuate inflammation. Alternatively, *GZMB* may have noncytolytic functions such as inducing proinflammatory cytokine release, as has been demonstrated for *GZMA* (32). Additional analyses of gene expression changes downstream of the predicted ligand-receptor interactions revealed enrichment of pathways including IL-7 signaling in healthy CD8^+^ T_EM_ cells (Figure 3b, left); by contrast, CD8^+^ T_EM_ cells derived from patients with CD exhibited enrichment of pathways including CD28 costimulation, IL-2 signaling, and IFN-γ signaling (Figure 3b, right), along with upregulation of the cytolytic molecule *GZMA* and the long noncoding RNA *NEAT1* (Figure 3c), which has suggested to regulate T-cell apoptosis and cytolytic activity (33).

We next examined the group of cells that included both CD4^+^ and CD8^+^ T_RM_ cells along with MAIT cells. As with ileal CD8^+^ T_EM_ cells, we observed that healthy T_RM_/MAIT cells interacted with a broad array of immune cells, whereas ileal T_RM_/MAIT cells derived from patients with CD exhibited increased interactions with innate immune cells, including monocytes, myeloid dendritic cells, and ILC/NK cells (see Supplementary Figure 3A, http://links.lww.com/CTG/A923). Moreover, differences observed with ileal CD8^+^ T_EM_ cells with respect to ligand-receptor interactions were also observed with ileal T_RM_/MAIT cells between the healthy and CD state, such as *TNFRSF14*-*TNFSF14*, *TNFRSF14-BTLA*, and *IL7-IL7R* in healthy subjects, and *GZMB*-*IGF2R*, *CXCL8*-*CXCR2*, and *IL1B*-*IL1R1* interactions in patients with CD (see Supplementary Figure 3B, http://links.lww.com/CTG/A923). However, 1 unique interaction not predicted in ileal CD8^+^ T_EM_ cells was *IL18* produced by myeloid dendritic cells binding to *IL18R* and *IL18RAP* in ileal T_RM_/MAIT cells (see Supplementary Figures 3A, 2B, http://links.lww.com/CTG/A923). IL-18 is an IL-1 family cytokine that can promote the production of IFNγ in T cells (34).

Analyses of potential gene expression changes downstream of predicted ligand-receptor interactions in healthy subjects revealed induction of *ABCB1*, *ZBTB16*, *IRF9*, and *IL7R* in healthy ileal T_RM_/MAIT cells (see Supplementary Figure 3C, left, http://links.lww.com/CTG/A923). *ABCB1* encodes a member of family of ATP-binding cassette transporters that transfer various molecules across membranes (35). *ZBTB16* is the transcription factor associated with MAIT cells, but is also expressed by a number of tissue-resident and unconventional T cells (36), whereas *IRF9* is a component of type I interferon signaling downstream of the IFN-I receptor (37). Analyses of gene expression changes downstream of predicted ligand-receptor interactions in patients with CD revealed induction of the inflammatory cytokines *IFNG* and *IL17A*, along with several transcription factors: *BHLHE40*, *ZFP36*, and *EGR1* (see Supplementary Figure 3C, right, http://links.lww.com/CTG/A923). *BHLHE40* is a transcription factor that has been implicated as a molecular switch among CD4^+^ T-cell fates, such as Th1, Th2, and Th17 (38–40), as well as a regulator of mitochondrial fitness and the epigenetic state in CD8^+^ T_RM_ cells (41). *EGR1* regulates T-cell development, activation, and proliferation (42), and *ZFP36* encodes for tristetraprolin, an RNA-binding protein that has been reported to be a negative regulator of differentiation and cytotoxicity of CD8^+^ T cells (43).

**Figure 4.**
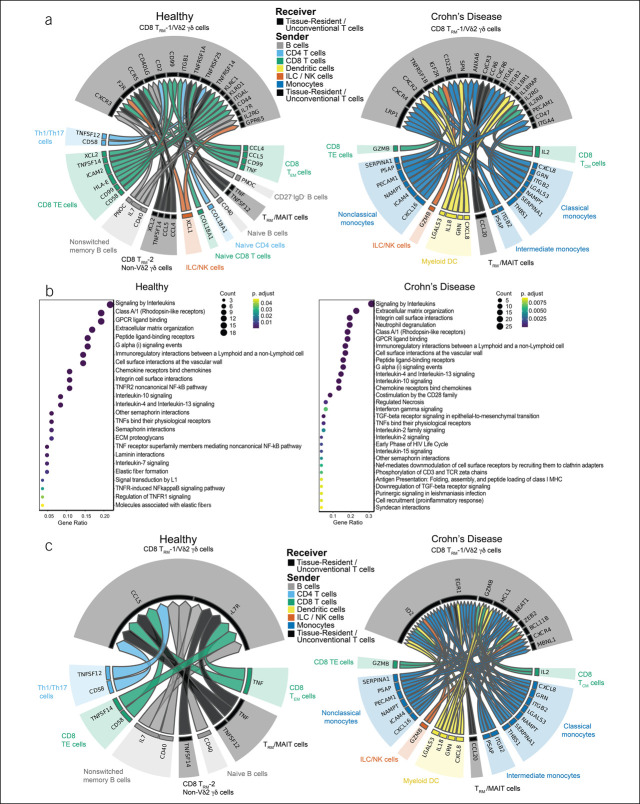
NicheNet analyses of ileal CD8 T_RM_-cell interactions with other immune cells in health vs Crohn's disease (CD). (**a**) Putative ligand:receptor pairs on “sender” immune cells and receiver cell (CD8 T_RM_-1/Vδ2 γδ T cells), represented as Circos plots, in healthy subjects (left) or patients with CD (right). Colors indicate major immune cell type; each major cell type is also indicated in the caption between the 2 Circos plots. Specific immune cell subsets are labeled around the Circos plot. (**b**) Pathway analyses of enriched genes downstream of predicted ligand:receptor pairs, represented as reactome plots, in healthy subjects (left) or patients with CD (right). (**c**) Genes predicted to be induced in CD8 T_RM_-1/Vδ2 γδ T cells by predicted ligand:receptor interactions, represented as Circos plots, in healthy subjects (left) or patients with CD (right).

Finally, we examined 2 groups of cells that included CD8^+^ T_RM_ and γδ T cells, annotated as CD8^+^ T_RM_-1/Vδ2 γδ T cells (Figure 4) and CD8^+^ T_RM_-2/non-Vδ2 γδ T cells (see Supplementary Figure 4, http://links.lww.com/CTG/A923). As with other T-cell subsets described above, both groups of CD8^+^ T_RM_/γδ T cells exhibited diverse interactions with other immune subsets in health, whereas increased interactions with innate immune cells were predicted in CD (Figure 4a, see Supplementary Figure 4A, http://links.lww.com/CTG/A923). Moreover, interactions such as *IL7*-*IL7R* and *CCL5*-*CXCR3* were again predicted in the healthy state, whereas in CD, *GZMB*-*IGF2R*, *IL18*-*IL18R1*, and *IL2*-*IL2RB* interactions along with downstream signaling pathways were again predicted to be enriched (Figure 4a,b, see Supplementary Figures 4A, 4B, http://links.lww.com/CTG/A923). In addition, several unique interactions were predicted. For example, TNF produced by multiple cellular sources was predicted in the healthy state for both groups of cells, indicating a counterintuitive role for TNF in health and cell homeostasis (Figure 4a,b, see Supplementary Figures 4A, 4B, http://links.lww.com/CTG/A923).

Analyses of gene expression changes downstream of predicted ligand-receptor interactions in health revealed induction of *CCL5* and *IL7R* in CD8^+^ T_RM_-1/Vδ2 γδ T cells (Figure 4c, left) and FBJ murine osteosarcoma viral oncogene homolog and avian sarcoma virus 17 oncogene homolog, members of the activator protein-1 family of transcription factors, in CD8^+^ T_RM_-2/non-Vδ2 γδ T cells (see Supplementary Figure 4C, left, http://links.lww.com/CTG/A923). Analyses of gene expression changes downstream of predicted ligand-receptor interactions in patients with CD revealed induction of *ID2*, *EGR1*, *GZMB*, *MCL1*, *BCL11B*, *NEAT1*, and *ZEB2* in CD8^+^ T_RM_-1/Vδ2 γδ T cells (Figure 4c, right) and *NEAT1*, *GZMB*, *PRF1*, and *ZFP36* in CD8^+^ T_RM_-2/non-Vδ2 γδ T cells (see Supplementary Figure 4C, right, http://links.lww.com/CTG/A923). *ZFP36* encodes for a zinc finger motif-containing Kruppel-type zinc finger protein previously reported to influence Th2 cell differentiation in allergic airway inflammation (44). *MCL1* encodes for an antiapoptotic protein, which promotes cell survival (45). *BCL11B* encodes for a well-established transcription factor that regulates T-cell developmental fate decisions (46), but has also been shown to regulate CD8^+^ T-cell expansion and cytolytic function (47). *ID2* encodes a transcriptional regulator that negatively regulates the E2A protein and promotes effector CD8 T-cell differentiation (48,49), whereas *ZEB2* encodes a transcriptional repressor that also promotes terminal effector CD8^+^ T-cell differentiation (50–52). Taken together, these findings identify putative cell-cell interactions and transcriptional consequences that may lead to some of the changes observed in IBD.

## DISCUSSION

In this study, we performed CITE-seq to simultaneously measure the transcriptome and a panel of proteins in the same single cells derived from the peripheral blood, colons, and ileums of healthy subjects and individuals with ileal CD. We focused our NicheNet analyses on CD8^+^ T-cell subsets to identify interacting immune cells, along with specific ligand-receptor pairs and key transcriptional changes resulting from these CCC. We observed that in the context of CD, ileal CD8^+^ T_EM_ and T_RM_ cells interacted to a greater extent with monocyte subsets and myeloid dendritic cells (Figure 3a, right). These observations extend the findings of Martin et al, who previously identified a unique cellular module in patients with CD consisting of IgG plasma cells, inflammatory mononuclear phagocytes, activated T cells, and stromal cells (12).

We identified cytokine signaling pathways that have been previously implicated in IBD, including those downstream of IL-1β, IL-18, and IL-17A. We also identified a number of putative ligand-receptor pairs and signaling pathways that have been less well-studied in IBD. One example is ALCAM, expressed on nonclassical monocytes and myeloid dendritic cells and predicted to bind to CD6, a costimulatory receptor expressed by T cells, which has been shown to regulate T-cell activation, proliferation, and trafficking (30,31). Compared with control mice, CD6-deficient mice exhibited less severe disease in several murine autoimmune models, including experimental autoimmune encephalomyelitis, psoriasis, and uveitis (53–55). Consistent with these findings, a humanized anti-CD6 monoclonal antibody, itolizumab, has been shown to be effective for the treatment of psoriasis and rheumatoid arthritis (56,57), owing to its ability to inhibit T-cell activation, proliferation, and production of proinflammatory cytokines (58,59), and is being studied as a therapy for acute graft-vs-host disease (60). Intriguingly, *CD6* is a risk susceptibility gene in IBD (61), and a higher proportion of CD4^+^ T cells derived from intestinal tissues from patients with IBD expressed CD6 compared with healthy subsets (62), indicating that CD6 could represent a potential therapeutic target in IBD.

An example of a signaling pathway not been previously linked to IBD was the cytolytic molecule granzyme B. Granzyme B is perhaps best known for its role in CD8^+^ T-cell–mediated killing of infected cells during immune responses against microbial infections. In this study, we observed that in the context of CD, granzyme B produced by CD8^+^ T cells was predicted to act on other CD8^+^ T-cell subsets. As granzymes have been reported to have noncytolytic functions (63), such as inducing inflammatory cytokine release, this finding may represent a previously unappreciated mechanism by which granzymes promote inflammation in IBD. Indeed, granzyme A can induce the release of IL-8 by epithelial cells; IL-6 and IL-8 by fibroblasts; and IL-1β, TNF, IL-6, and IL-8 by human monocytes (64), at least *in vitro*. Moreover, granzyme B can convert pro–IL-18 into active IL-18 as well as cleave the precursor of IL-1α, thereby increasing its biologic activity (63). Alternatively, the prediction that granzyme B acts directly on CD8^+^ T cells in CD may represent a compensatory mechanism by which CD8^+^ T cells can attenuate the damage and inflammation caused by cytolytic cells in the setting of IBD. Indeed, some cytolytic molecules required by effector CD8^+^ T cells to eliminate infected cells during microbial infection have been shown to play a role to control the size of the expanded CD8^+^ T-cell pool during and after infection. For example, perforin has been implicated in limiting the expansion and elimination of activated antigen-specific CD8^+^ T cells during chronic infection (65) and graft-vs-host disease (66) as well as after viral infection (67). These observations raise the possibility that targeting production of cytolytic molecules may be of therapeutic benefit in IBD.

The study is not without limitations. The study included a small number of healthy individuals and patients with ileal CD, which limits its potential generalizability. CCC involving key nonimmune cells known to play a role in IBD, such as epithelial and stromal cells, were not investigated. Furthermore, although we focused our analyses on signals received by CD8^+^ T-cell subsets, signals received by any immune cell subset in the data set could be interrogated. Finally, the predictions made by NicheNet are not unequivocal and represent a starting point for future validation and mechanistic studies. Nonetheless, the data set adds to the single-cell data available for human IBD and represents a resource for future research by other investigators. Overall, our work highlights the potential value of elucidating specific CCC in advancing our mechanistic understanding of IBD.

## CONFLICTS OF INTEREST

**Guarantor of the article:** John T. Chang, MD.

**Specific author contributions:** All authors significantly participated in the drafting of the manuscript or critical revision of the manuscript and provided approval of the final submitted version. B.S.B. and J.T.C.: conceptualized the study. H.G.D., E.C., N.R.C., S.A.P., J.G.O., Y.C.L., Y.H.L, P.Y., P.H., W.H.W., C.S.I., M.S.T., and B.S.B.: performed analysis or investigation. W.W. and J.T.C.: provided supervision of the study.

**Financial support:** CITE-seq using the 10x Genomics platform was performed at the UCSD IGM Genomics Center and supported by NIH grants P30KC063491, P30CA023100, and S10OD026929. This work was supported by the NIDDK-funded San Diego Digestive Diseases Research Center (P30DK120515) and funded by grants from the NIH DK007202 (M.S.T.); DK123406 (B.S.B.); AI129973, AI123202, BX005106, and CX002396 (J.T.C.); and AI132122 (W.W. and J.T.C.).

**Potential competing interests:** B.S.B. has received institutional research grants from Prometheus Biosciences and Gilead and has received institutional consulting fees from Bristol Myers Squibb, Takeda, and Pfizer. J.T.C. has received research grants from Takeda and Eli Lilly. All other authors declare that the research was conducted in the absence of any commercial or financial relationships that could be construed as a potential conflict of interest.

**Study Highlights:**

**WHAT IS KNOWN**

✓ Crohn’s disease (CD) is associated with dysregulated innate and adaptive immune responses to gut microbiota
✓ Heterogenous cell types have been implicated in CD pathogenesis, but the specific cell-cell communications involved remain incompletely characterized

**WHAT IS NEW HERE**

✓ Signaling to CD8 T cell subsets from other immune cell types differs in patients with CD compared to healthy subjects
✓ Interleukin-1β, interleukin-18, and granzyme B may be associated with CD8 T cell subsets in patients with CD

## Supplementary Material

SUPPLEMENTARY MATERIAL
