# Supplementary material for: Identification of CD8+ T-Cell–Immune Cell Communications in Ileal Crohn's Disease
Source: Clin Transl Gastroenterol. 2023 Mar 1;14(5):e00576. doi: 10.14309/ctg.0000000000000576 (PMC10208704; doi:10.14309/ctg.0000000000000576)
Supplement: SUPPLEMENTARY MATERIAL [file ct9-14-e00576-s003.doc]

Supplementary Material

# Supplementary Figures
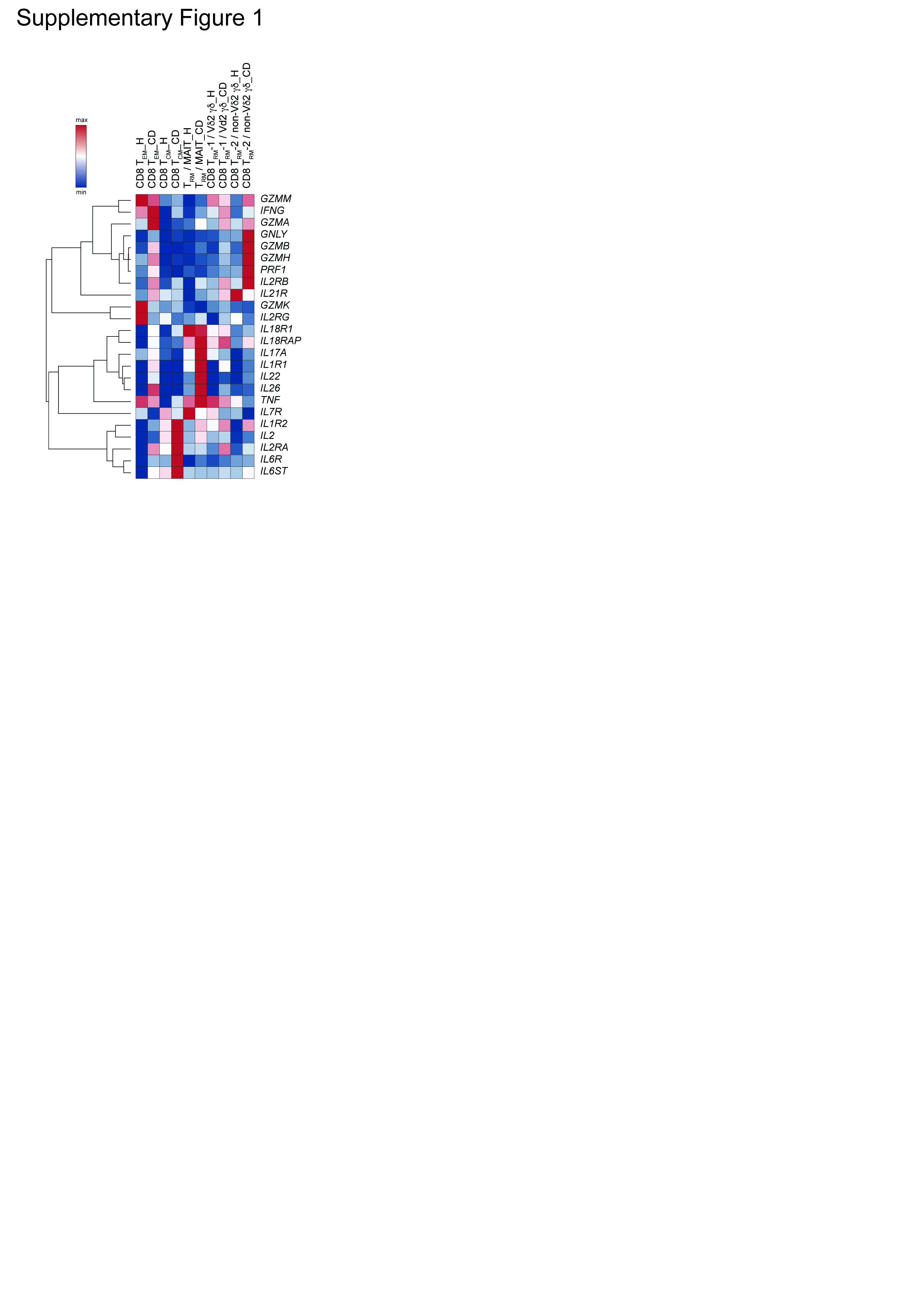


# Supplementary Figure 1. Expression of selected genes among CD8^+^ T cell subsets from healthy subjects vs. CD patients. Relative expression of selected genes in CD8^+^ T cell subsets from healthy (H) subjects vs. CD patients (CD), represented as hierarchically clustered summary heatmaps, with rows representing selected genes and columns representing CD8^+^ T cell subsets.

**
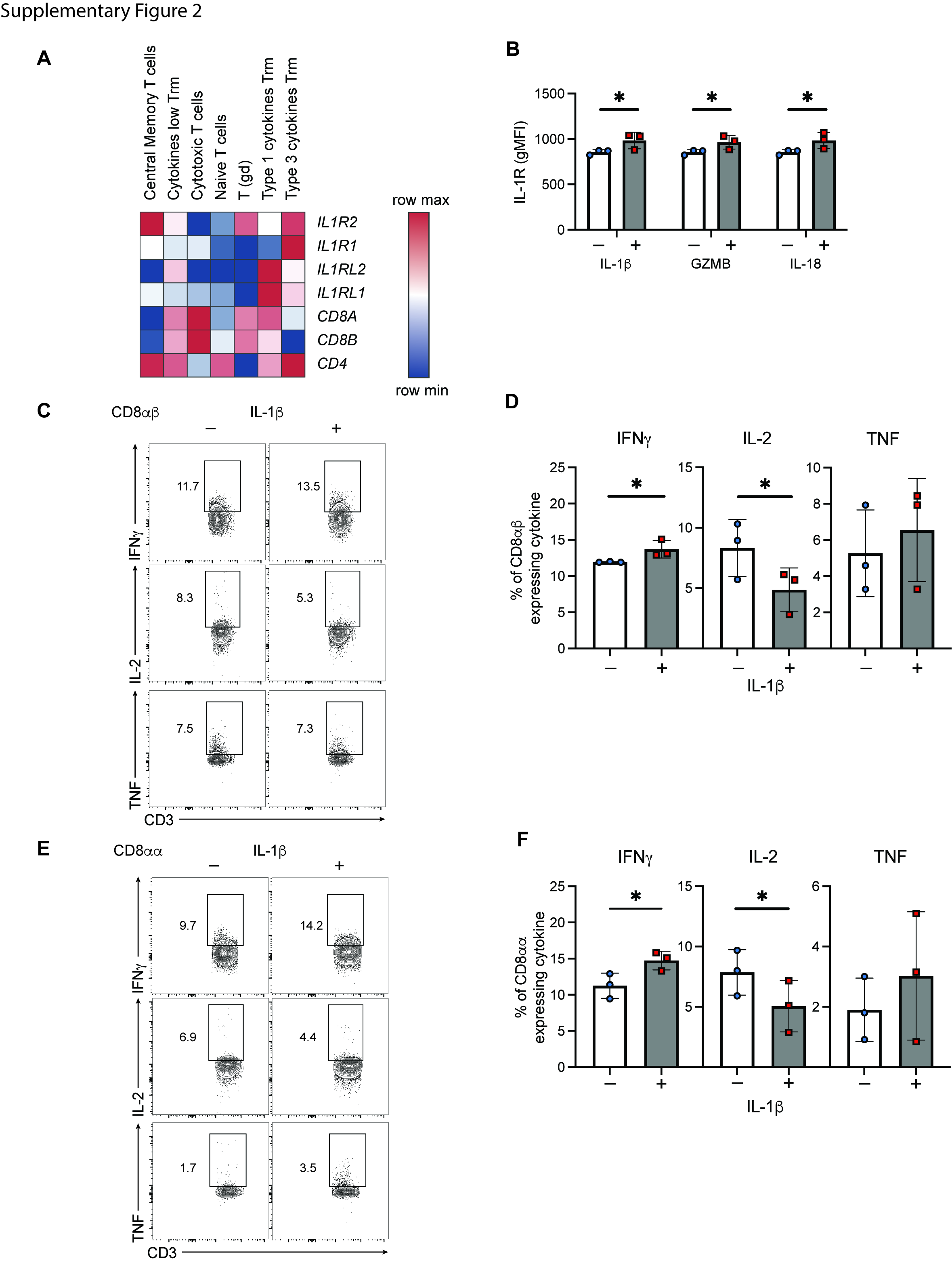
Supplementary Figure 2. Cytokine production in CD8 T cells modulated by interleukin-1β (IL-1β).** (A) Gene expression of IL-1 family members in T cell subsets derived from Martin et al., *Cell* 2019, represented as a summary heatmap. Small intestine cells isolated from 3 individual mice were treated with 1 ng/mL of IL-1β, granzyme B (GZMB), interleukin-18 (IL-18), or vehicle control in the presence of phorbol myristate acetate, ionomycin, brefeldin A, and monensin for 4 hours. (B) Geometric mean fluorescence intensity (gMFI) of IL-1R among small intestine CD8 lymphocytes (n = 3, * *p* < 0.05, 2-way ANOVA). (C) Representative flow plots showing interferon-γ (IFNγ), interleukin-2 (IL-2), or tumor necrosis factor (TNF) production by CD3^+^CD8αα^+^ T lymphocytes. (D) Quantification of the percentage of live CD3^+^CD8αα^+^ T lymphocytes expressing cytokines, represented as a bar plot (n = 3, * *p* < 0.05, 2-way ANOVA). (E) Representative flow plots showing IFNɣ, IL-2, and TNF production by CD3^+^CD8αβ^+^ T lymphocytes. (F) Quantification of the percentage of live CD3^+^CD8αβ^+^ T lymphocytes expressing cytokines, represented as a bar plot (n = 3, * *p* < 0.05, 2-way ANOVA).

**
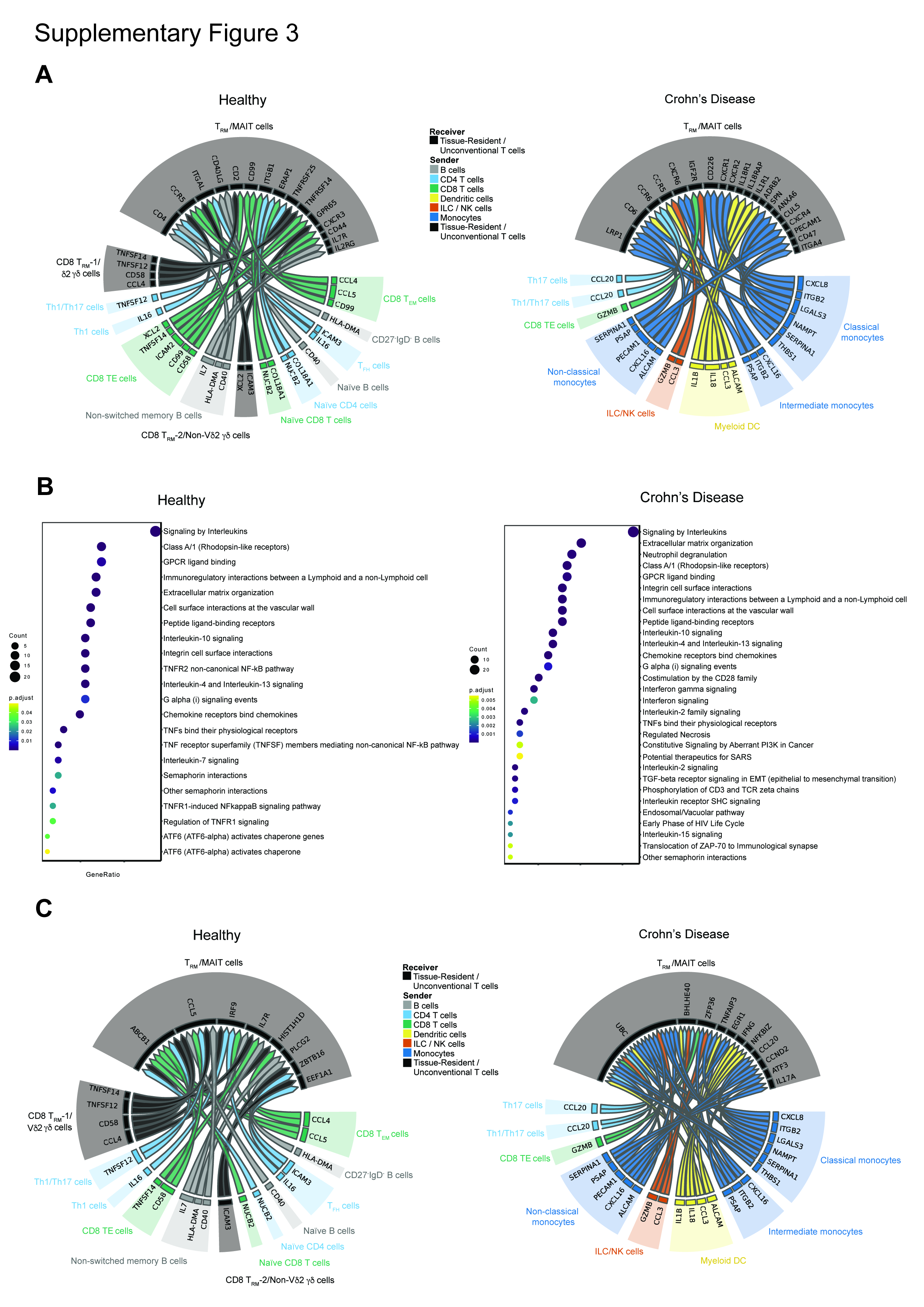
Supplementary Figure 3. NicheNet analyses of ileal T_RM_/MAIT cell interactions with other immune cells in health vs. Crohn’s disease**. (**a**) Putative ligand: receptor pairs on ‘sender’ immune cells and ‘receiver’ cells (T_RM_/MAIT cells), represented as Circos plots, in healthy subjects (left) or CD patients (right). Colors indicate major immune cell type; each major cell type is also indicated in the caption between the two Circos plots. Specific immune cell subsets are labeled around the Circos plot. (**b**) Pathway analyses of enriched genes downstream of predicted ligand:receptor pairs, represented as reactome plots, in healthy subjects (left) or CD patients (right). (**c**) Genes predicted to be induced in T_RM_/MAIT cells by predicted ligand:receptor interactions, represented as Circos plots, in healthy subjects (left) or CD patients (right).

**
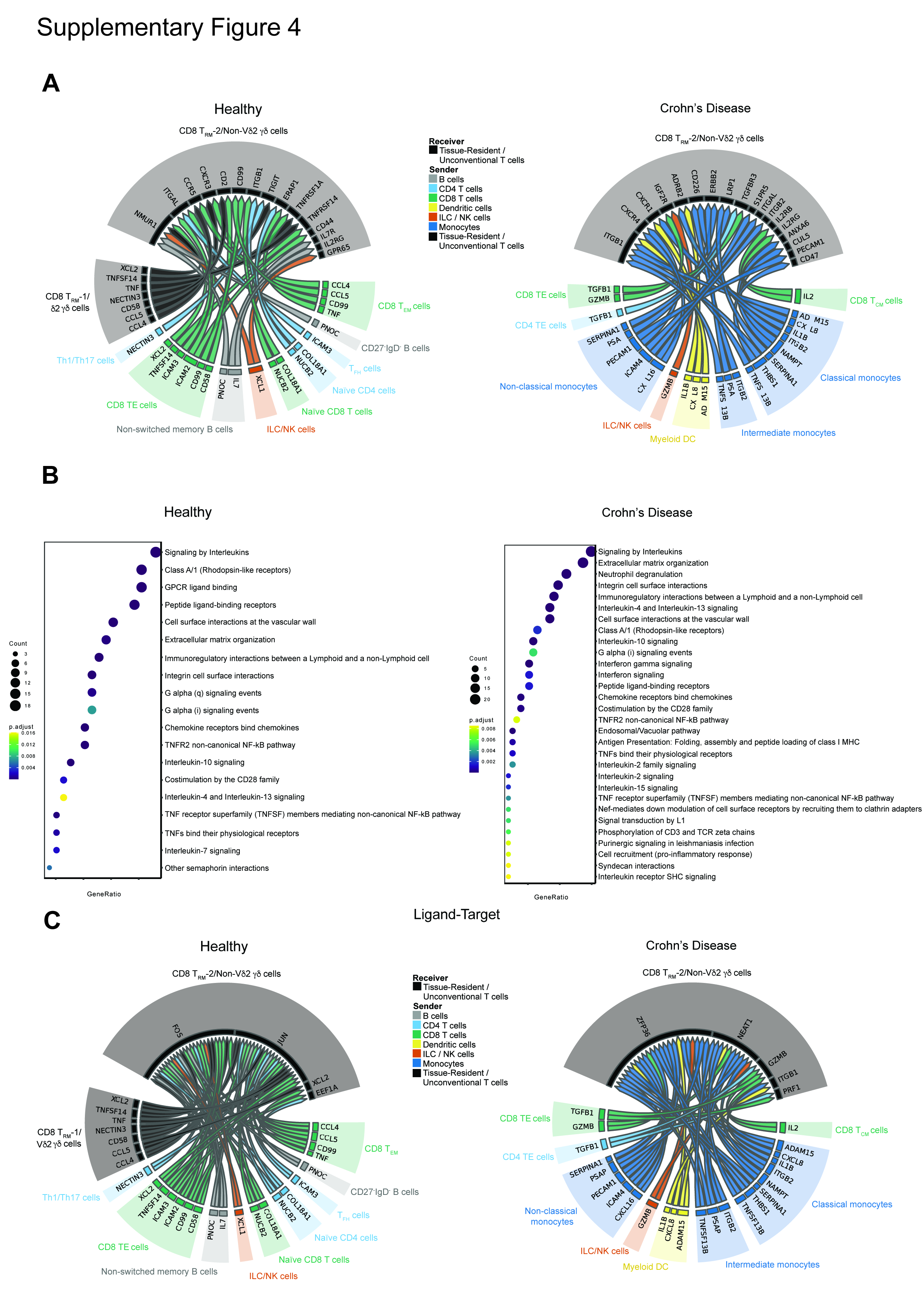
Supplementary Figure 4. NicheNet analyses of ileal CD8 T_RM_-2/non-Vδ2 γδ T cell interactions with other immune cells in health vs. Crohn’s disease**. (**a**) Putative ligand: receptor pairs on ‘sender’ immune cells and ‘receiver’ cells (CD8 T_RM_-1/non-Vδ2 γδ T cells), represented as Circos plots, in healthy subjects (left) or CD patients (right). Colors indicate major immune cell type; each major cell type is also indicated in the caption between the two Circos plots. Specific immune cell subsets are labeled around the Circos plot. (**b**) Pathway analyses of enriched genes downstream of predicted ligand:receptor pairs, represented as reactome plots, in healthy subjects (left) or CD patients (right). (**c**) Genes predicted to be induced in CD8 T_RM_-1/non-Vδ2 γδ T cells by prioritized ligand:receptor interactions, represented as Circos plots, in healthy subjects (left) or CD patients (right).
