# Supplementary material for: Identification of CD8+ T-Cell–Immune Cell Communications in Ileal Crohn's Disease
Source: Clin Transl Gastroenterol. 2023 Mar 1;14(5):e00576. doi: 10.14309/ctg.0000000000000576 (PMC10208704; doi:10.14309/ctg.0000000000000576)
Supplement: SUPPLEMENTARY MATERIAL [file ct9-14-e00576-s004.pdf]

Supplementary Material

Supplementary Figures

Supplementary Figure 1

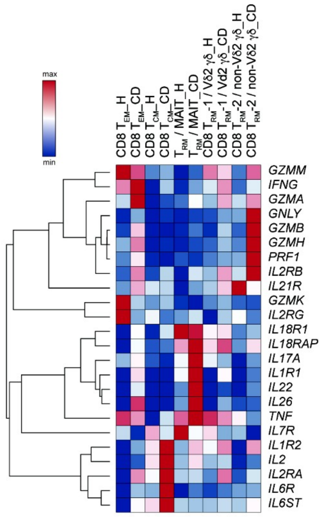

Supplementary Figure 1. Expression of selected genes among CD8$^+$ T cell subsets from healthy subjects vs. CD patients. Relative expression of selected genes in CD8$^+$ T cell subsets from healthy (H) subjects vs. CD patients (CD), represented as hierarchically clustered summary heatmaps, with rows representing selected genes and columns representing CD8$^+$ T cell subsets.

Supplementary Material

Supplementary Figure 2

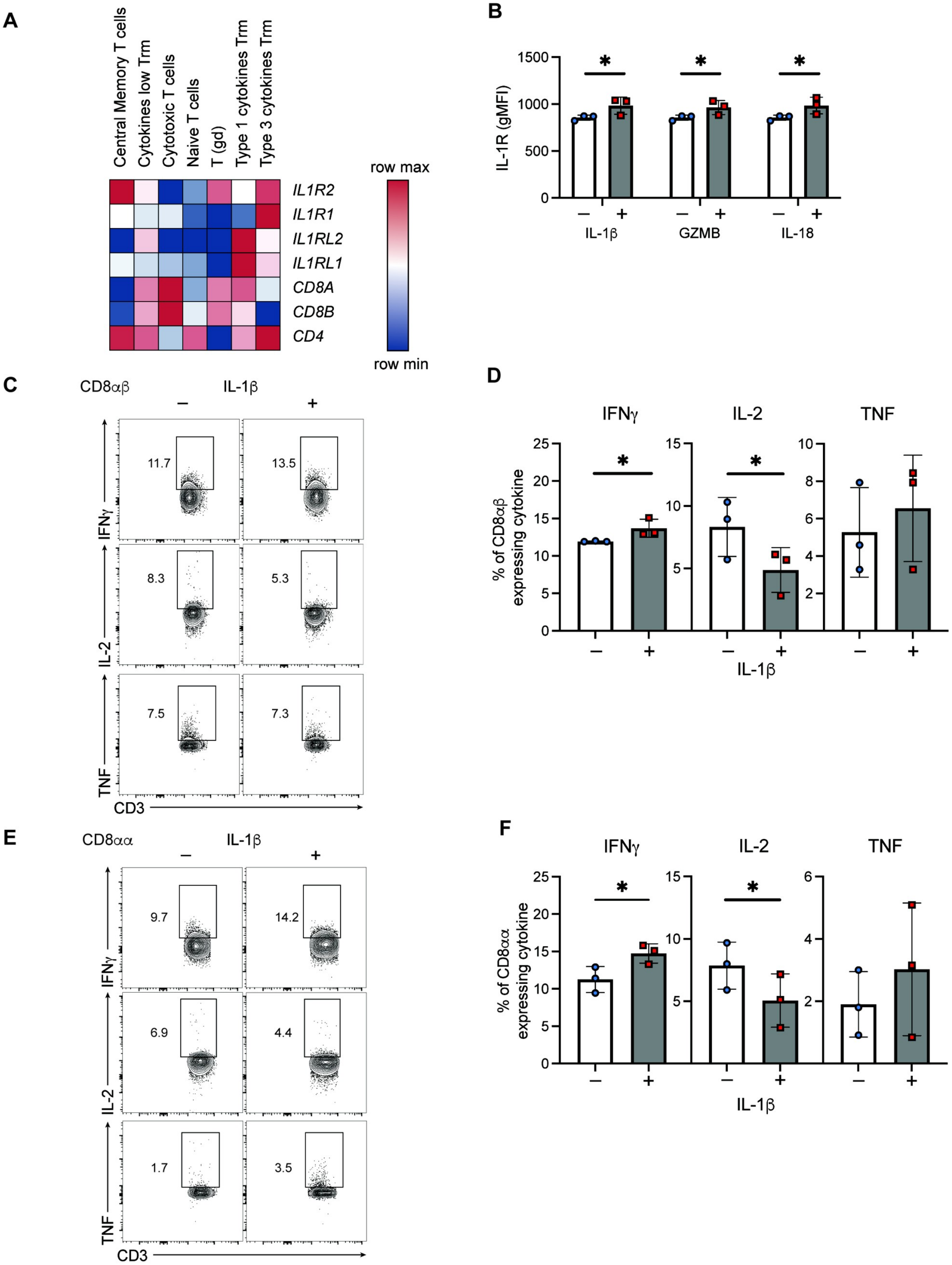

Supplementary Figure 2. Cytokine production in CD8 T cells modulated by interleukin-1β (IL-1β). (A) Gene expression of IL-1 family members in T cell subsets derived from Martin et al., *Cell* 2019, represented as a summary heatmap. Small intestine cells isolated from 3 individual mice were treated with 1 ng/mL of IL-1β, granzyme B (GZMB), interleukin-18 (IL-18), or vehicle control in the presence of phorbol myristate acetate, ionomycin, brefeldin A, and monensin for 4 hours. (B) Geometric mean fluorescence intensity (gMFI) of IL-1R among small intestine CD8 lymphocytes (n = 3, * $p < 0.05$, 2-way ANOVA). (C) Representative flow plots showing interferon-γ (IFNγ), interleukin-2 (IL-2), or tumor necrosis factor (TNF) production by $CD3^{+}CD8\alpha\alpha^{+}$ T lymphocytes. (D) Quantification of the percentage of live $CD3^{+}CD8\alpha\alpha^{+}$ T lymphocytes expressing cytokines, represented as a bar plot (n = 3, * $p < 0.05$, 2-way ANOVA). (E) Representative flow plots showing IFNɣ, IL-2, and TNF production by $CD3^{+}CD8\alpha\beta^{+}$ T lymphocytes. (F) Quantification of the percentage of live $CD3^{+}CD8\alpha\beta^{+}$ T lymphocytes expressing cytokines, represented as a bar plot (n = 3, * $p < 0.05$, 2-way ANOVA).

Supplementary Material

Supplementary Figure 3

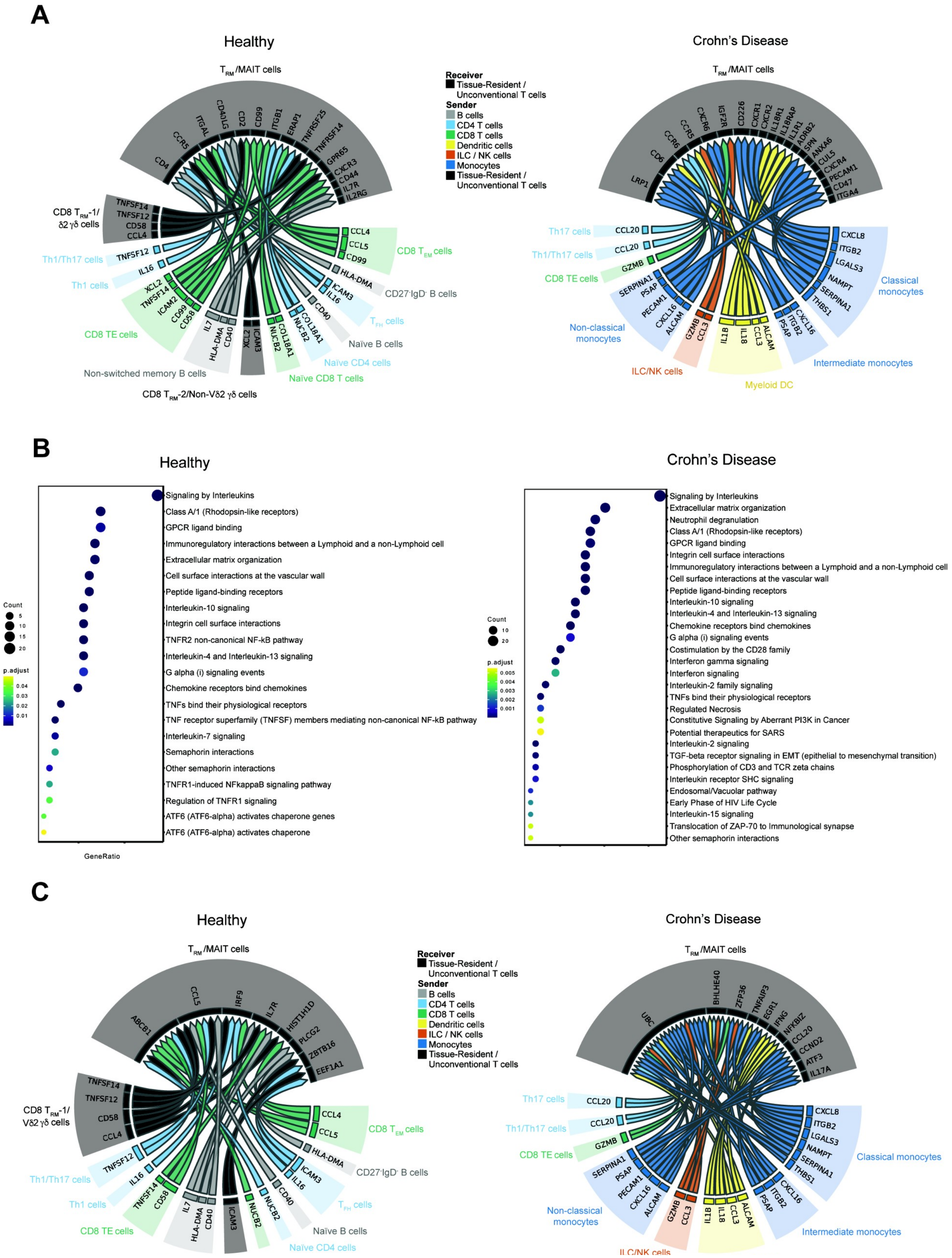

**upplementary Figure 3. NicheNet analyses of ileal TRM/MAIT cell interactions with other immune cell
s in health vs. Crohn's disease.** (a) Putative ligand: receptor pairs on 'sender' immune cells and 'recei
ver' cells (TRM/MAIT cells), represented as Circos plots, in healthy subjects (left) or CD patients (ri
ght). Colors indicate major immune cell type; each major cell type is also indicated in the caption b
etween the two Circos plots. Specific immune cell subsets are labeled around the Circos plot
. (b) Pathway analyses of enriched genes downstream of predicted ligand:receptor pairs, represented as re
actome plots, i n healthy subjects (left) or CD patients (right). (c) Genes predicted to be induced in
TRM/MAIT cells by predicted ligand:receptor inter

Supplementary Figure 4

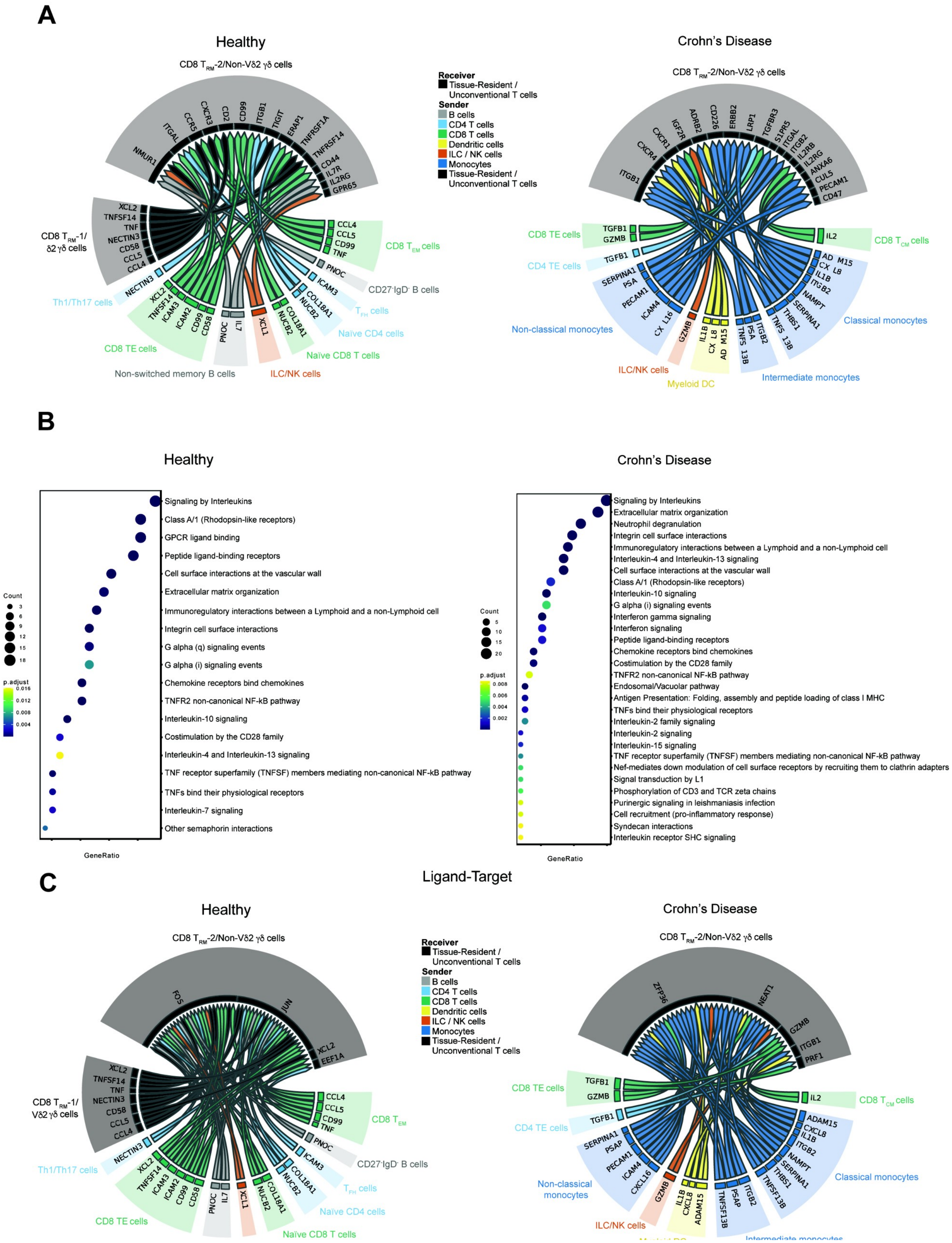

**upplementary Figure 4. NicheNet analyses of ileal CD8TR$_{M}$-2/non-Vd2 gd T cell interactions wit
h other immune cells in health vs. Crohn's diseaes** (a) Put ati ve ligand: receptor pairs on 'sende
r' immune cells and 'receiver' cells (CD8 TRM-1/non-Vd2 gd T cells), represented as Circos plots, in he
althy subjects (left) or CD patients (right). Colors indicate major immune cell type; each major cel
l type is also indicated in the caption betwe en the two Circos plots. Specific immune cell subset
s are labeled around the Circos plot. (b) Pathway analyses of enriched genes downstream of predic
ted ligand:receptopairs, represented as reactome plots, in h ealthy s ubjects (left) or CD patients
(right). (c) Genes predicted to be induced in CD8 TRM-1/non-Vd2 gd T cells by prioritized ligand:rec
eptor in
